# Supplementary material for: SCIG: Machine learning uncovers cell identity genes in single cells by genetic sequence codes
Source: Nucleic Acids Res. 2025 May 28;53(10):gkaf431. doi: 10.1093/nar/gkaf431 (PMC12117433; doi:10.1093/nar/gkaf431)
Supplement: gkaf431_Supplemental_Files [file gkaf431_supplemental_files.zip › SCIG_Supplementary_Figures1-10.pdf]

Supplementary Figures

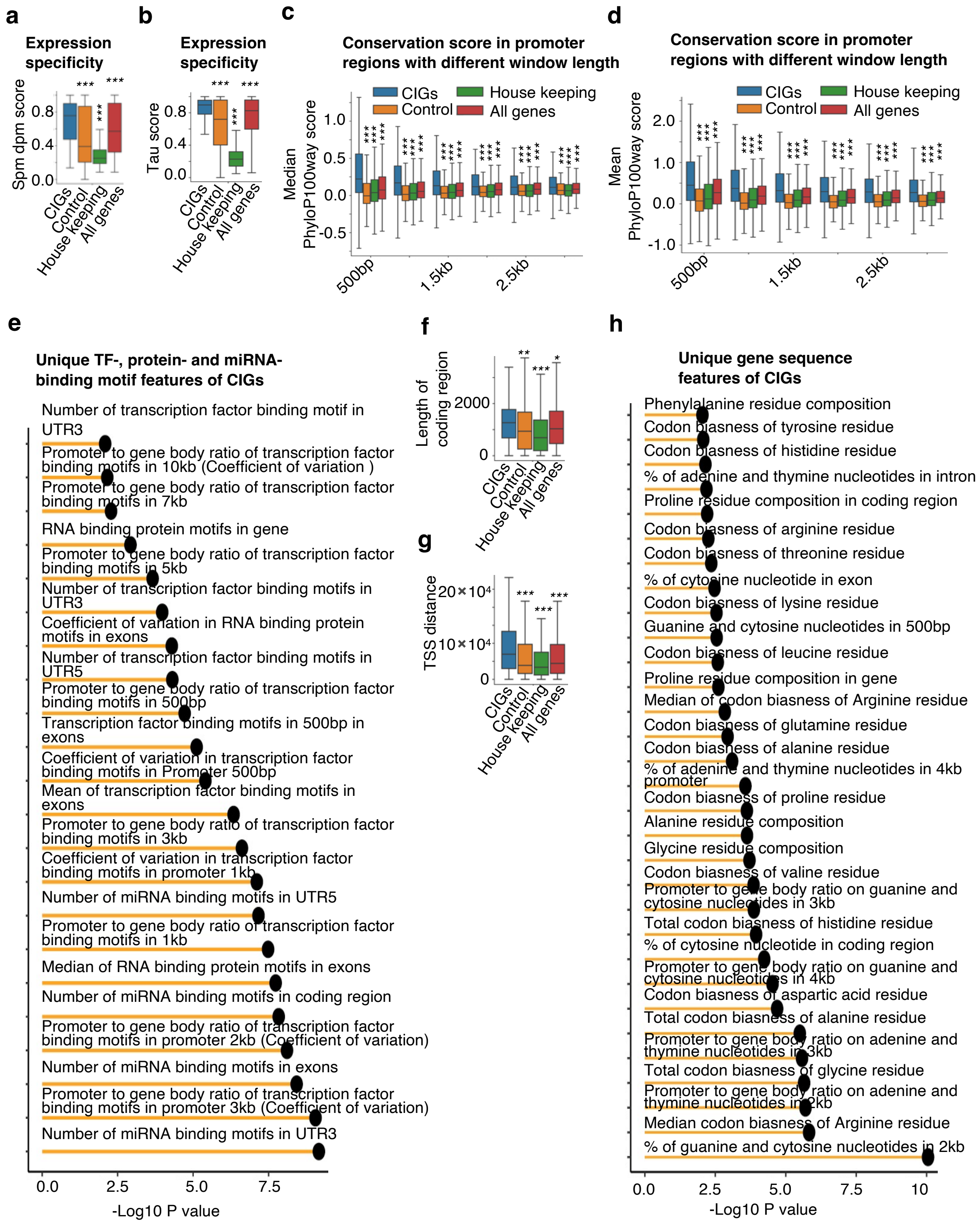

Figure S1. Distinctive genetic sequence signatures and expression features distinguish cell identity genes from other genes. (a-b) Box plot showing expression specificity determined by specificity measure dispersion (spm dpm) (**a**) and Tau score (**b**) for individual gene categories. **(c-d)** Median (**c**) and mean (**d**) PhyloP100way scores for promoters of individual gene categories. Scores calculated based on different promoter sizes, defined as different lengths flanking the transcription start sites, were presented. **(e)** Bar plot showing the significance of difference in individual motif features between CIGs and control genes. **(f-g)** Boxplot showing length of coding regions (**f**) and neighbor TSS distance for individual gene groups. **(h)** Bar plot showing the significance of difference in individual gene sequence features between CIGs and control genes. The two-tailed-Wilcoxon test was performed to determine the P-value of significance. *, p-value < 0.05; **, p-value < 0.01; ***, p-value < 0.001.

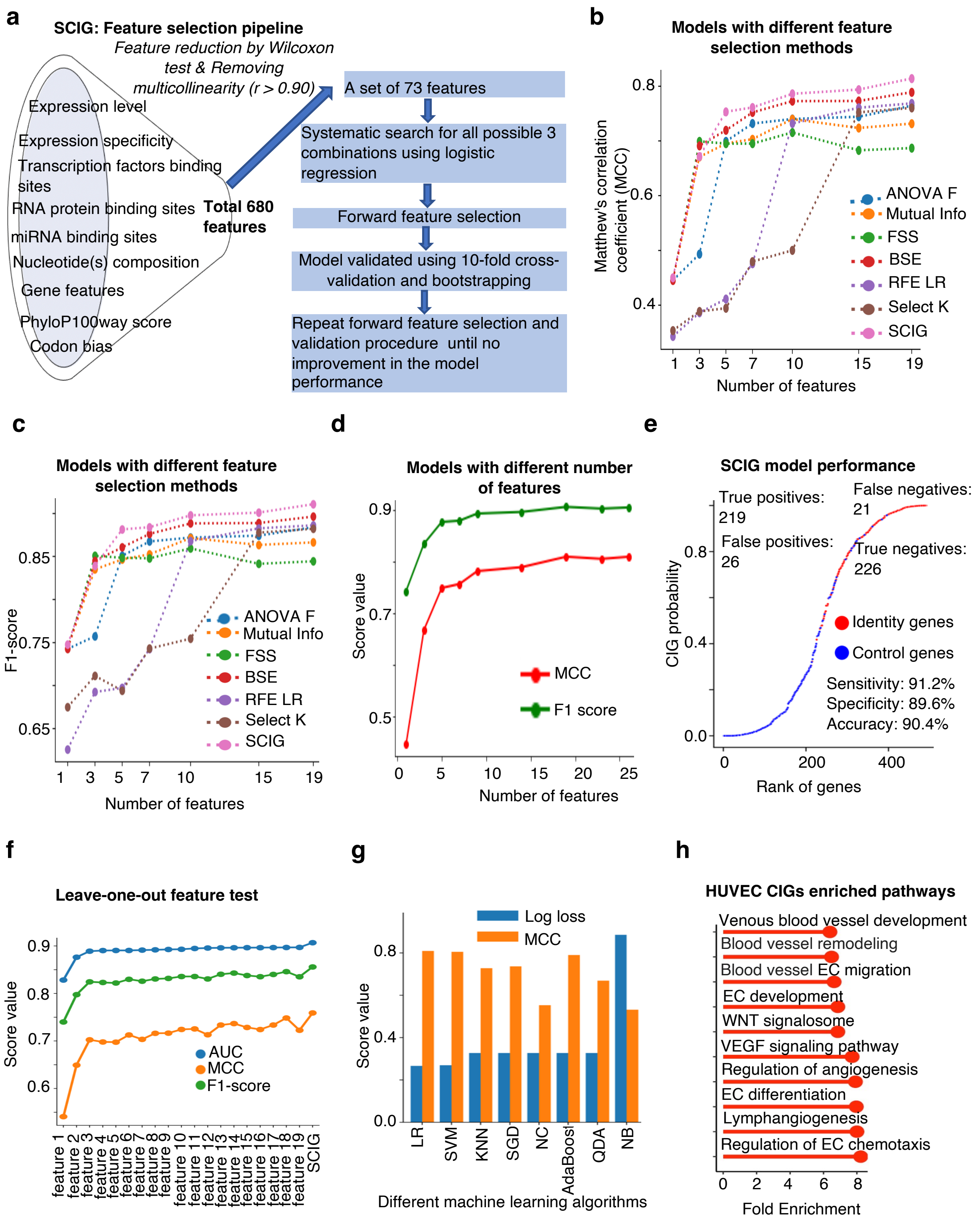

Figure S2. SCIG is a robust machine-learning algorithm for uncovering cell identity genes. **(a)** Workflow illustrating the process of feature selection in SCIG. **(b-c)** Line plot depicting correlation coefficient **(b)** and F1 score **(c)** as measurements of the performance of different feature selection methods, including sklearn Analysis of variance derived F-value (ANOVA F),

mutual information (MI), forward sequential selection (FSS), backward stepwise elimination (BSE), recursive feature elimination based on Logistic Regression (RFE LR), and SelectKBest (Select K). **(d)** Line plot depicting the performance of SCIG variants trained with different numbers of features. **(e)** Rank plot demonstrating the CIG probability of individual genes. **(f)** Performance of SCIG variants that were each trained with one of the 19 features depleted for comparison to the SCIG trained with all 19 features. X-axis indicates the individual depleted features. **(g)** Bar plots showing the performance of alternative machine learning algorithms to uncover CIGs based on genetic sequence signatures and gene expression features. *LR, Logistic Regression; SVM, support vector machine; KNN, K-Nearest Neighbour; SGD, Stochastic gradient descent; NC, Nearest centroid; LDA, Linear Discriminant Analysis; AdaBoost, Adaptive Boosting; NB, Naive Bayes.* **(h)** Bar plot showcasing pathway enrichment of CIGs uncovered by SCIG in HUVEC. EC, Endothelial cells; WNT, wingless-related integration site; VEGF, vascular endothelial growth factor.

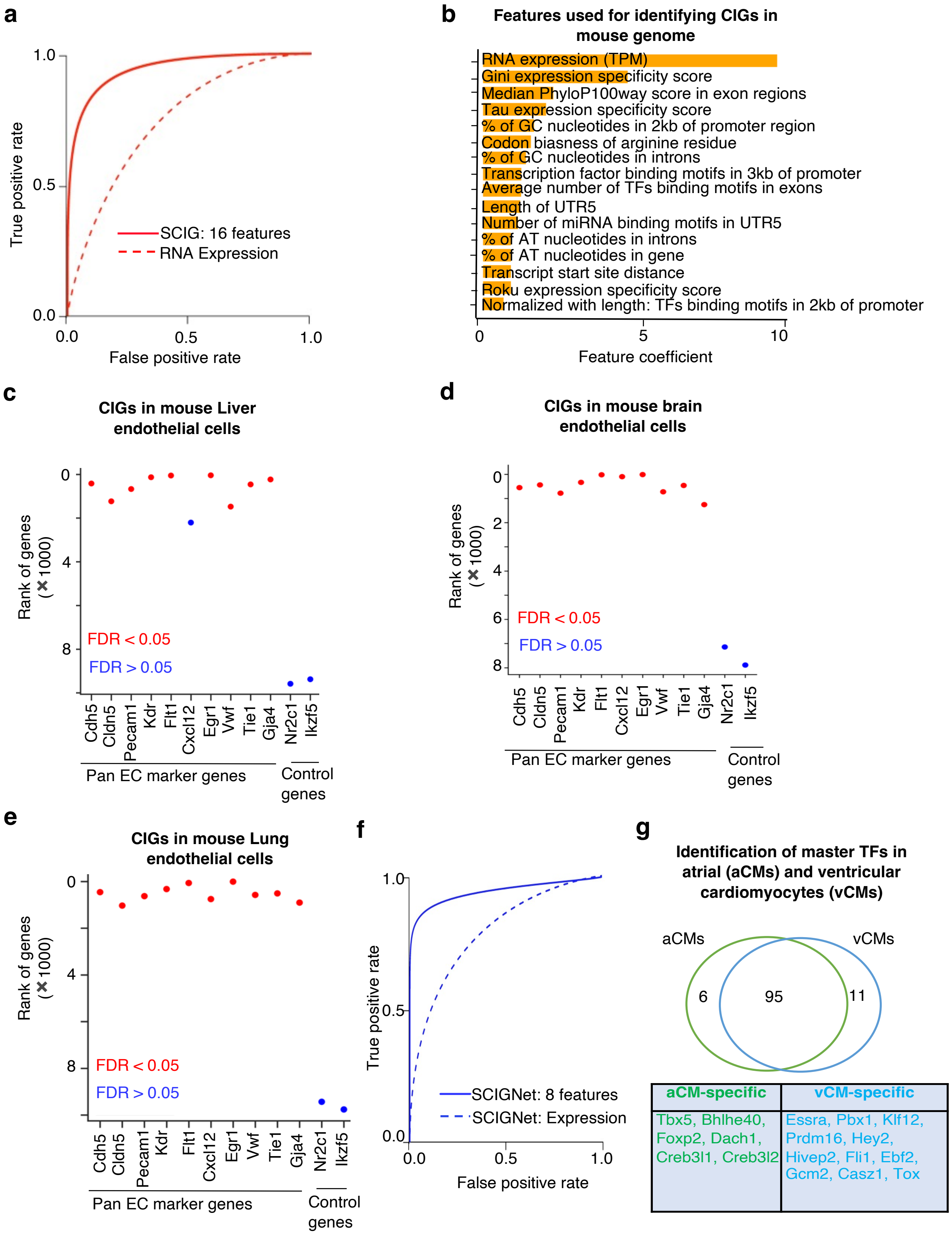

a
1.0
0.5
0.0
True positive rate
SCIG: 16 features
RNA Expression
0.0
0.5
1.0
False positive rate
b
Features used for identifying CIGs in mouse genome
RNA expression (TPM)
Gini expression specificity score
Median PhyloP100way score in exon regions
Tau expression specificity score
% of GC nucleotides in 2kb of promoter region
Codon biasness of arginine residue
% of GC nucleotides in introns
Transcription factor binding motifs in 3kb of promoter
Average number of TFs binding motifs in exons
Length of UTR5
Number of miRNA binding motifs in UTR5
% of AT nucleotides in introns
% of AT nucleotides in gene
Transcript start site distance
Roku expression specificity score
Normalized with length: TFs binding motifs in 2kb of promoter
0
5
10
Feature coefficient
c
CIGs in mouse Liver endothelial cells
Rank of genes (✖1000)
0
4
8
FDR < 0.05
FDR > 0.05
Cdh5
Cldn5
Pecam1
Kdr
Flt1
Cxcl12
Egr1
Vwf
Tie1
Gja4
Nr2c1
Ikzf5
Pan EC marker genes
Control genes
d
CIGs in mouse brain endothelial cells
Rank of genes (✖1000)
0
2
4
6
8
FDR < 0.05
FDR > 0.05
Cdh5
Cldn5
Pecam1
Kdr
Flt1
Cxcl12
Egr1
Vwf
Tie1
Gja4
Nr2c1
Ikzf5
Pan EC marker genes
Control genes
e
CIGs in mouse Lung endothelial cells
Rank of genes (✖1000)
0
4
8
FDR < 0.05
FDR > 0.05
Cdh5
Cldn5
Pecam1
Kdr
Flt1
Cxcl12
Egr1
Vwf
Tie1
Gja4
Nr2c1
Ikzf5
Pan EC marker genes
Control genes
f
1.0
0.5
0.0
True positive rate
SCIGNet: 8 features
SCIGNet: Expression
0.0
0.5
1.0
False positive rate
g
Identification of master TFs in atrial (aCMs) and ventricular cardiomyocytes (vCMs)
aCMs
vCMs
6
95
11
aCM-specific
vCM-specific
Tbx5, Bhlhe40, Foxp2, Dach1, Creb3l1, Creb3l2
Essra, Pbx1, Klf12, Prdm16, Hey2, Hivep2, Fli1, Ebf2, Gcm2, Casz1, Tox

Figure S3. SCIG uncovered cell identity genes in mouse cells with great accuracy.
(a) ROC curve illustrating the performance of SCIG in mouse cells with 16 genetic sequence and RNA expression features. **(b)** Bar plot showing importance 16 significant features in the prediction of CIGs in mouse cells. **(c-e)** Endothelial marker genes ranked by CIG scores calculated by SCIG in ECs from different mouse tissue types. **(f)** ROC curve illustrating the performance of SCIGNet in uncovering the master regulators of CIGs in mouse cells. **(g)** Venn diagram showing aCM-specific, vCM-specific, and common CM identity regulators uncovered by the SCIG model.

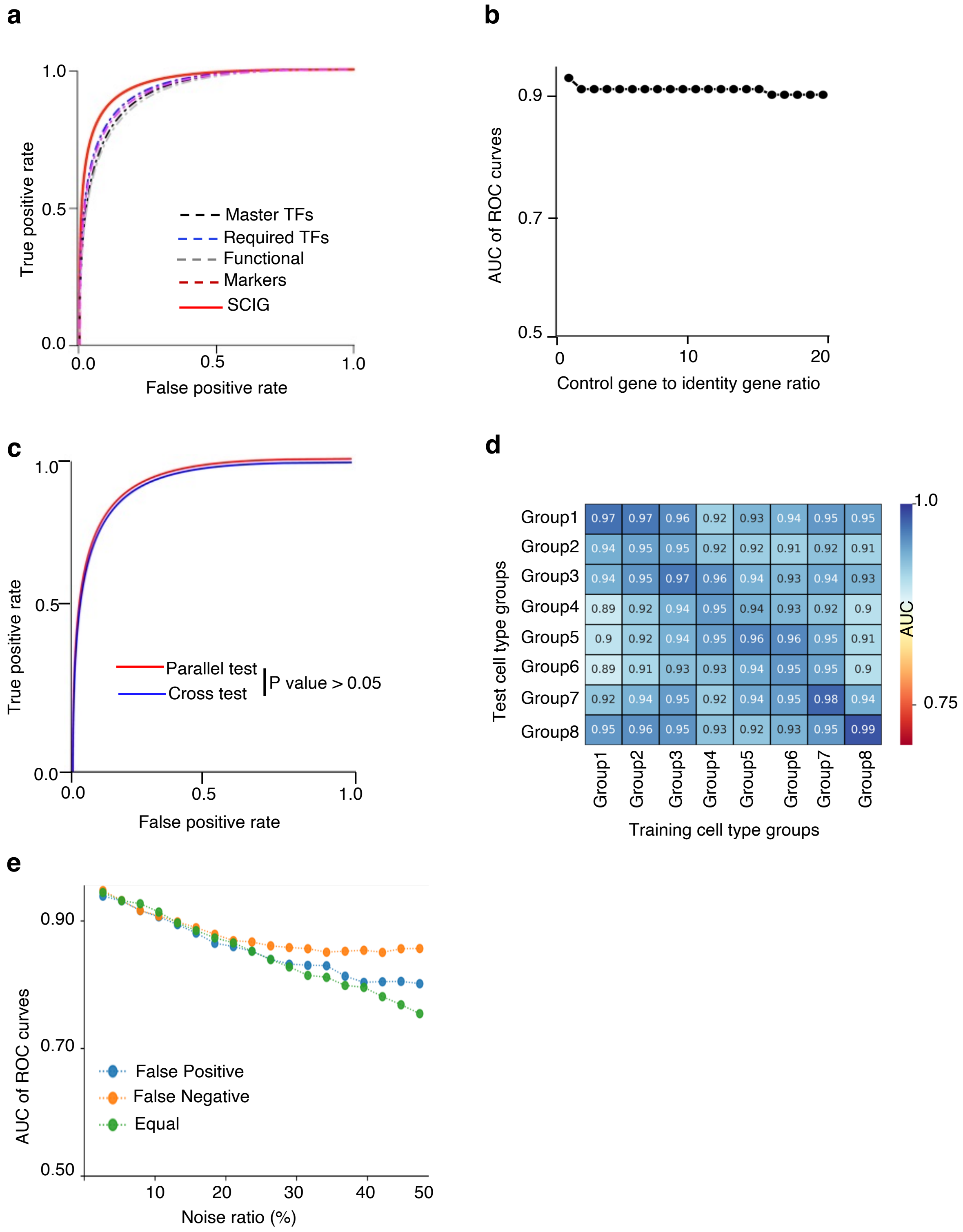

Figure S4. Robustness and noise tolerance analysis of SCIG.
(a) ROC curves demonstrating the performance of SCIG variants trained using different CIG categories but tested with all categories. **(b)** AUC plotted against the control gene to CIGs ratio in the training data for SCIG. The ratio is systematically increased by adding more control genes

while maintaining the number of CIGs constant. **(c)** ROC curves illustrating the performance of SCIG in both parallel test and cross test. In the parallel test, SCIG was trained and tested by data from the same cell types. In the cross test, SCIG was trained by data from one set of cell types and tested by data from a different set of cell types. **(d)** Heatmap showing the performance of models trained and tested with the same (parallel) and different (cross) groups of cell types. Note: Each group contains three cell types. The diagonal blocks represent the AUC values of models trained and tested with the same groups of cell types. Other blocks show models trained and tested with different cell types. **(e)** AUC plotted against the simulated noise ratio in the training data for SCIG. Different methods of noise introduction are tested. These include the False Positive method, in which negative control genes are swapped to identity genes; the False Negative method, in which identity genes are swapped to negative control genes; and the Equal method, in which an equal number of identity genes are swapped to control genes and control genes are swapped to identity genes.

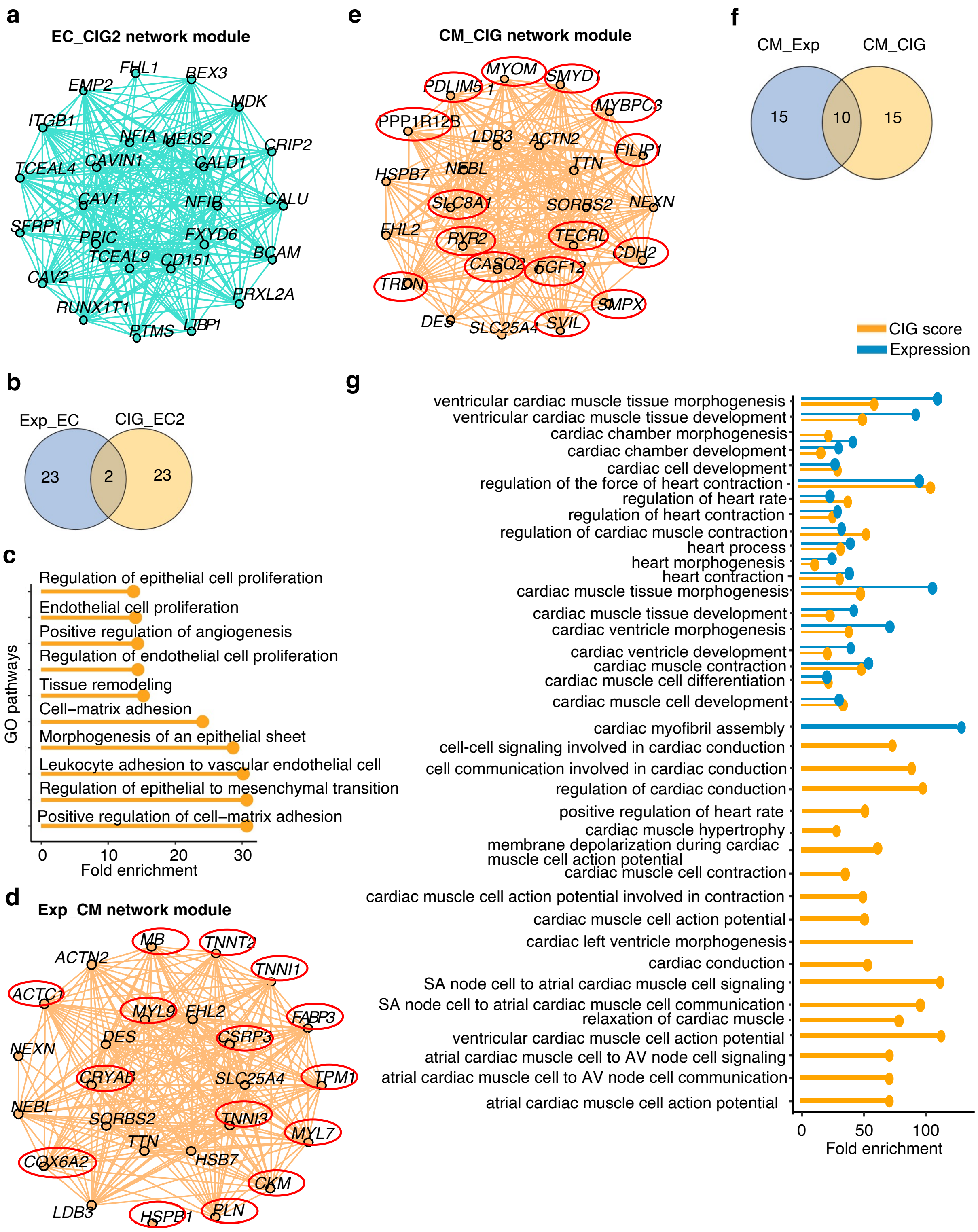

Figure S5. Application of SCIG to gene network analysis using hdWGCNA. (a) Network plot showing the top 25 hub genes of network module EC_CIG2 derived from CIG score. **(b)** Ven diagram showing the overlap of hub genes in CIG score-derived network model (EC_CIG2) and expression-derived network module (EC_Exp). **(c)** Pathway analysis of hub genes in the CIG

score-derived network module EC_CIG2. **(d)** Expression-derived cardiomyocytes network (CM_Exp) hub genes. **(e)** CIG score-derived cardiomyocytes network (CM_CIG) hub genes. **f)** Overlap of hub genes in cardiomyocytes network modules derived from expression and CIG score. **(g)** The CIG score-derived network hub genes are significantly enriched with cardiac pathways.

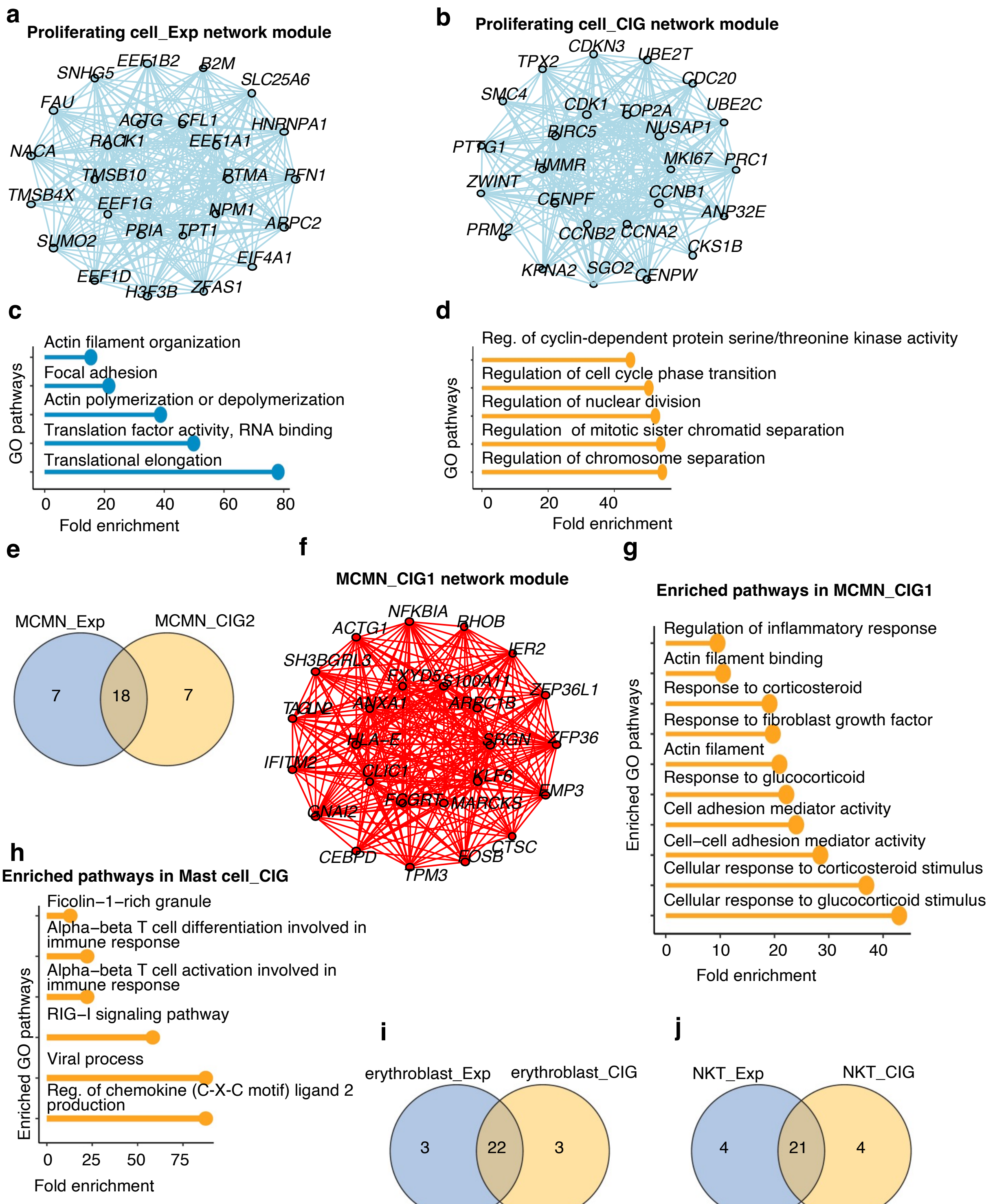

Figure S6. CIG Score Outperforms Expression Value in Capturing Cell Identity in Network Analysis. (a-b) Expression-derived (a) and CIG score-derived (b) gene network of proliferating cell. **(c-d)** Pathway analysis of hub genes from the expression-derived (c) and CIG score-derived (d) gene network of proliferating cell. **(e)** Venn diagram showing overlap between hub genes from the expression-derived and CIG score-derived gene network of the

macrophage/monocytes modules. **(f)** Network plot showing the hub genes of macrophages/monocytes gene network derived from the CIG score analysis. **(g-h)** Pathway analysis of hub genes in the macrophages/monocytes (g) and mast cell (h) gene networks derived from CIG score analysis. **(i-j)** Venn diagram showing overlap between hub genes between the expression- and CIG score-derived gene networks in the erythroblasts (i) and NKT (j) cells.

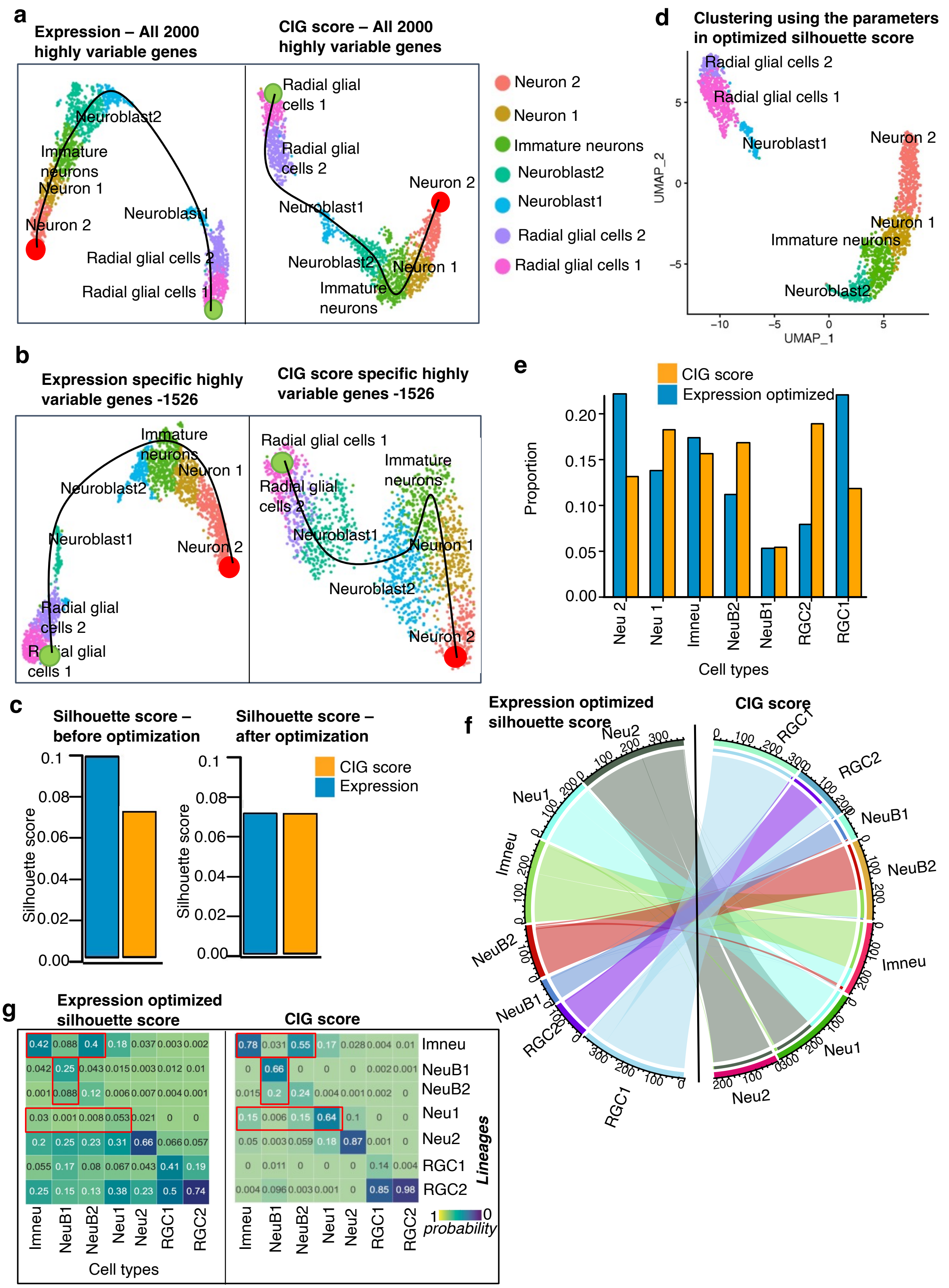
a
Expression – All 2000 highly variable genes
CIG score – All 2000 highly variable genes
Neuron 2
Neuron 1
Immature neurons
Neuroblast2
Neuroblast1
Radial glial cells 2
Radial glial cells 1
b
Expression specific highly variable genes -1526
CIG score specific highly variable genes -1526
c
Silhouette score – before optimization
Silhouette score – after optimization
Silhouette score
CIG score
Expression
d
Clustering using the parameters in optimized silhouette score
UMAP_1
UMAP_2
e
CIG score
Expression optimized
Proportion
Cell types
Neu 2
Neu 1
Imneu
NeuB2
NeuB1
RGC2
RGC1
f
Expression optimized silhouette score
CIG score
Neu2
Neu1
Imneu
NeuB2
NeuB1
RGC2
RGC1
g
Expression optimized silhouette score
CIG score
Imneu
NeuB1
NeuB2
Neu1
Neu2
RGC1
RGC2
Cell types
Lineages
probability

Figure S7. Cell Identity Score Improved Single-cell Trajectory Analysis of Neuronal Differentiation. **(a)** UMAP plot showing the identified cell clusters using top 2000 highly variable genes defined based on gene expression (left) or CIG scores (right). **(b)** UMAP plot showing the identified cell clusters using 1526 of the top 2000 highly variable genes defined based on gene expression (left) or CIG scores (right) after excluding the overlapped genes between the two lists. Green and red circles indicate locations of root and terminal cell types in trajectory analysis. The black lines indicate the differentiation trajectory. **(c)** Silhouette score for single-cell clustering based on CIG score or gene expression matrix before and after the optimization. **(d)** Single-cell clusters and cell type annotation based on gene expression matrix using fine-tuned clustering parameters (npcs = 70, dims = 1:70, resolution=1). **(e)** The proportion of cell types derived from clustering based on the CIG score or expression using fine-tuned clustering parameters. **(f)** The chord diagram displaying the label switching of individual cells clustered based on CIG score and the expression values (based on the fine-tuned clustering parameters). **(g)** Cellrank quantified the transition probability between cell populations defined based on CIG score or expression values, using the fine-tuned clustering parameters.

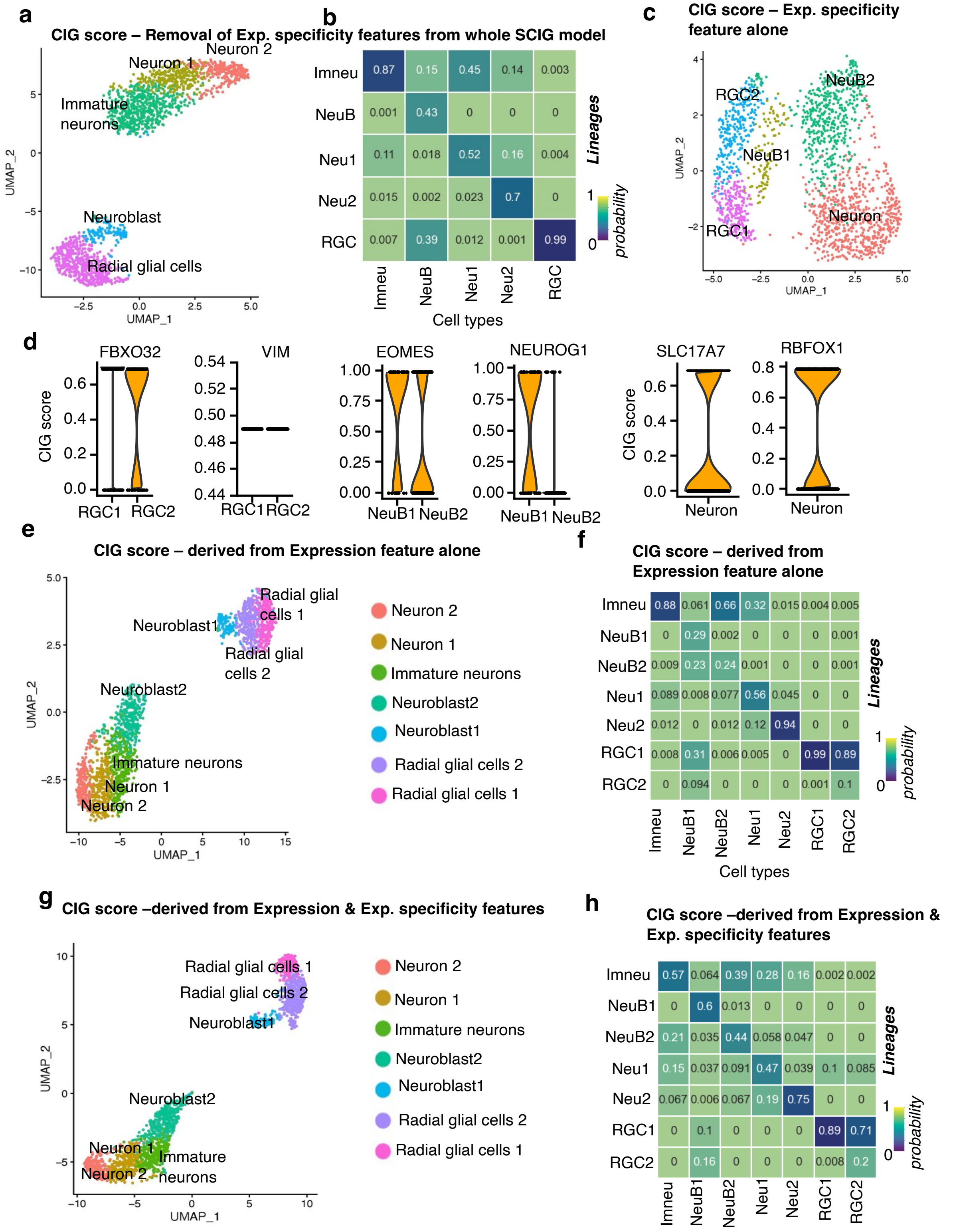

Figure S8: Contribution of gene expression and specificity features to SCIG prediction. **(a-b)** CIG scores computed for each single cell after removing the expression specificity features from

the whole SCIG model, along with their cell clustering and cell trajectory analysis. **(c)** CIG scores computed by including only the expression specificity features in the SCIG model, with cell clustering. **(d)** CIG scores predicted for genes using the expression specificity-based model. **(e-f)** CIG scores computed for each single cell by including only the expression features in the SCIG model, along with their cell clustering and cell trajectory analysis. **(g-h)** CIG scores computed for each single cell by including both expression and expression specificity features in the SCIG model, with corresponding cell clustering and cell trajectory analysis.

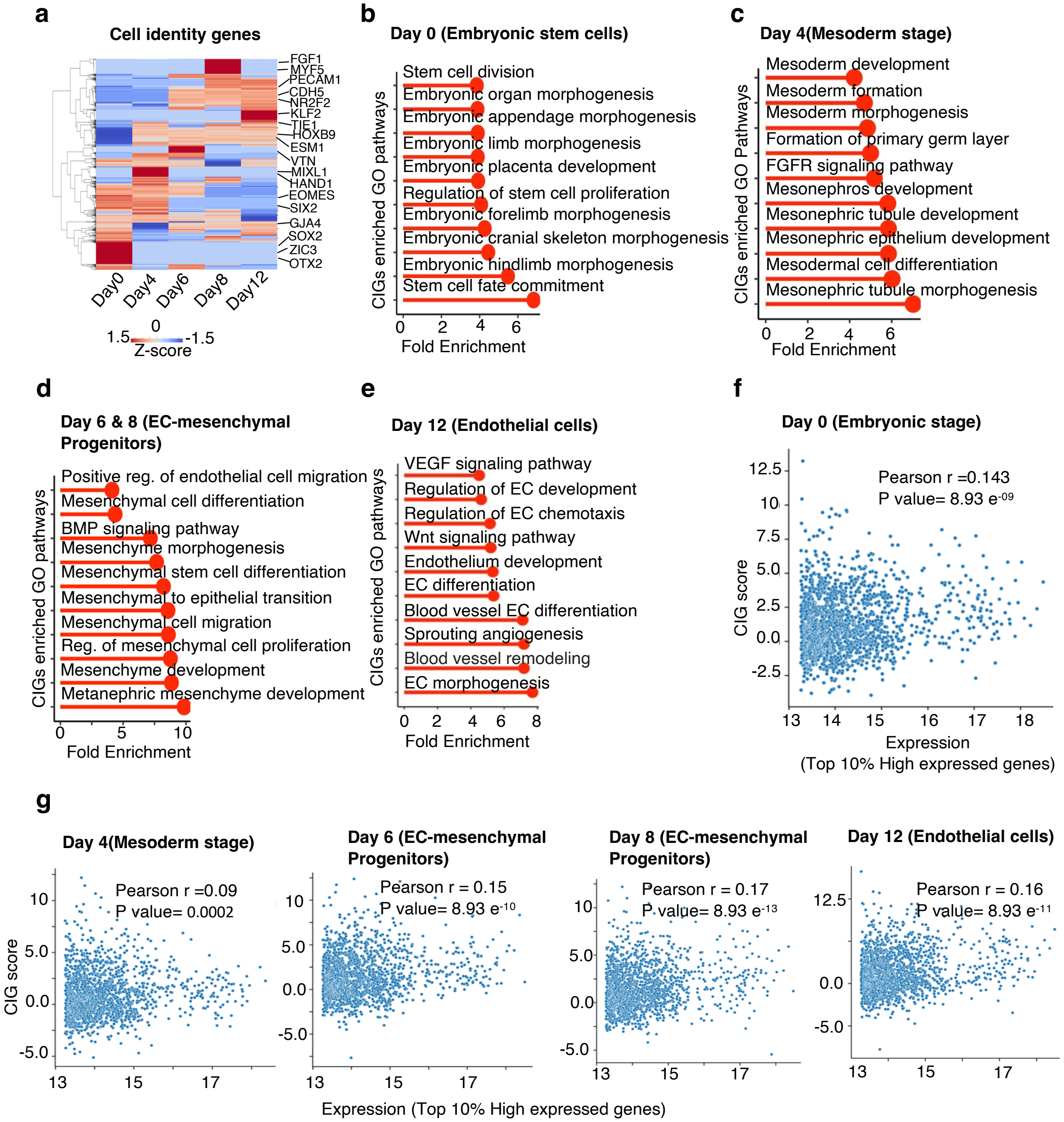

Figure S9. Cell identity gene (CIG) landscape in the process of endothelial differentiation from embryonic stem cells (ESC). (a) Heatmap displaying predicted cell identity genes across different stages of ESC to EC differentiation. **(b-e)** Pathway enrichment analysis of identified CIGs in ESC (b), Mesoderm (c), Progenitors (d), and endothelial (e) cells. **(f-g)** Scatter plot illustrating the correlation between expression values and predicted CIG scores of the top 10% highly expressed genes in embryonic stem cells (f) and individual differentiation stages (g).

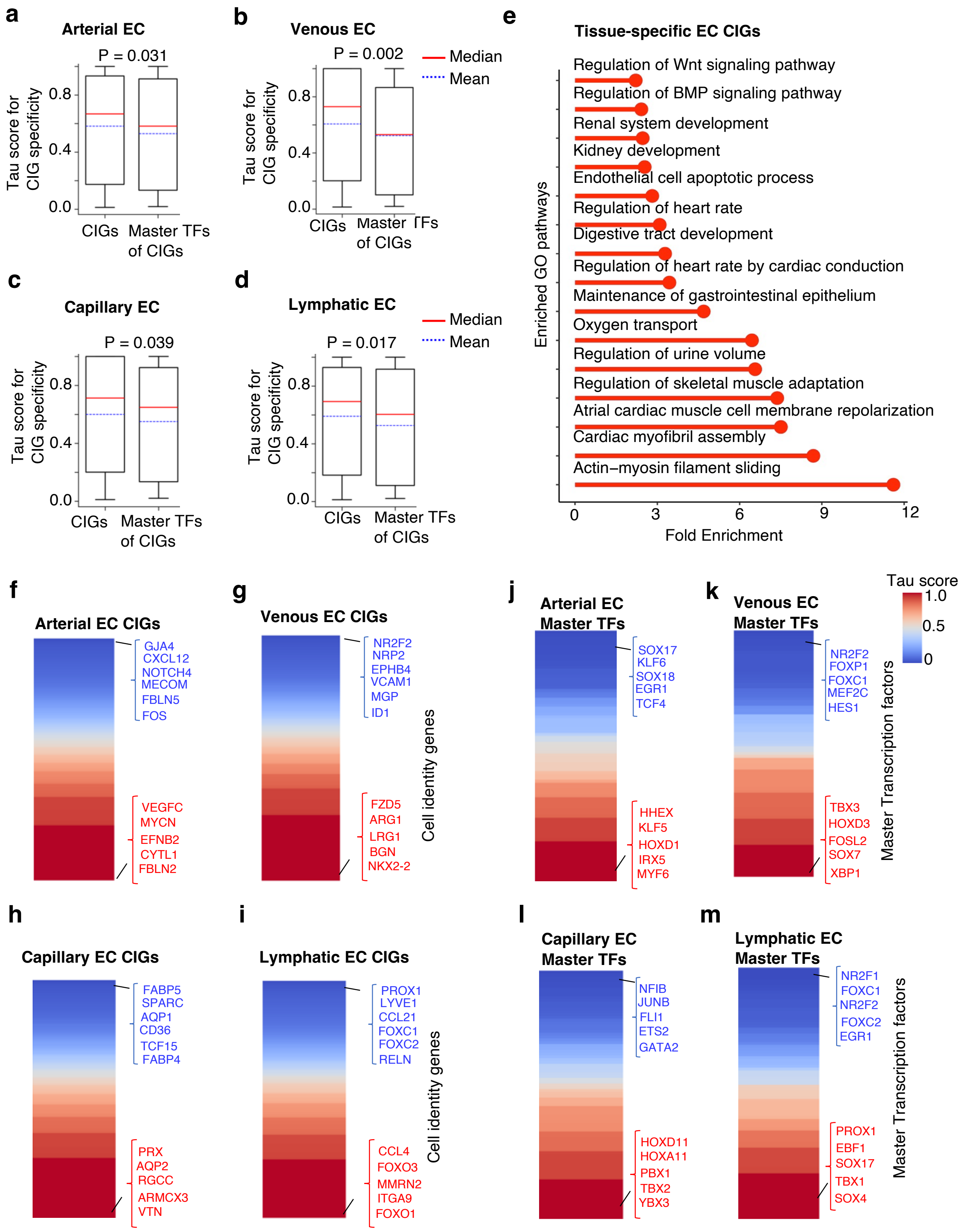

Figure S10. Landscape analysis of arterial, capillary, venous, lymphatic endothelial cell identity genes and their master transcription factors in 15 human tissues.
(a-d) Boxplot showing Tau score as a measurement of the expression specificity of CIGs and their regulators in individual endothelial subtypes across 15 tissues. **(e)** Pathway enrichment

analysis of tissue-specific endothelial CIGs. **(f-i)** Tau score computed for CIGs in individual EC subtypes across 15 tissue types. **(j-m)** Tau score computed for master transcription factors of CIGs in individual EC subtypes across 15 tissue types.
